# Supplementary material for: A protocol for a systematic review and meta-analysis of the effect of muscle energy techniques on shoulder joint pain
Source: PLoS One. 2025 Apr 17;20(4):e0321176. doi: 10.1371/journal.pone.0321176 (PMC12005804; doi:10.1371/journal.pone.0321176)
Supplement: S1 Appendix — (DOCX) [file pone.0321176.s001.docx]

**S1 Appendix. Search terms and strategies**

**Pubmed**

|  | Searches |
| --- | --- |
| #1 | “shoulder”[MeSH Terms] |
| #2 | “muscle energy technique”[MeSH Terms] |
| #3 | #1 AND #2 |

**EMBASE**

|  | Searches |
| --- | --- |
| #1 | ‘shoulder’/exp |
| #2 | ‘muscle energy technique’ /exp |
| #3 | #1 AND #2 |

**CENTRAL**

|  | Searches |
| --- | --- |
| #1 | [mh “shoulder”] |
| #2 | [mh “muscle energy technique”] |
| #3 | #1 AND #2 |

**Korean Medicla Database (KMbase)**

|  | Searches |
| --- | --- |
| #1 | Shoulder\|total |
| #2 | muscle energy technique\|total (((근에너지이완기법\|total) OR (근이완기법\|total)) OR (muscle energy technique\|total)) OR (MET\|total) |
| #3 | #1 AND #2 (muscle energy technique\|total) AND (shoulder\|total) |

**Korean Studies Information Service System (KISS)**

|  | Searches |
| --- | --- |
| #1 | ALL = “Shoulder” |
| #2 | ALL = "근에너지이완기법" or ALL = "근이완기법" or ALL = "muscle energy technique" or ALL = "MET" |
| #3 | #1 AND #2 |

**Research Information Service System (RISS)**

|  | Searches |
| --- | --- |
| #1 | ALL : shoulder |
| #2 | ALL : 근에너지이완기법 <OR> ALL : 근이완기법 <OR> ALL : muscle energy technique <OR> ALL : MET |
| #3 | #1 AND #2 |

**Korea Citation Index (KCI)**

|  | Searches |
| --- | --- |
| #1 | ALL : ‘shoulder’ |
| #2 | ALL : ‘근에너지이완기법 OR 근이완기법 OR (Muscle energy technique) OR MET' |
| #3 | #1 AND #2 |

**DBpia**

|  | Searches |
| --- | --- |
| #1 | ALL: “shoulder” |
| #2 | ALL: “근에너지이완기법”\|”근이완기법”\|"Muscle energy technique"\|”MET” |
| #3 | #1 AND #2 |
