# Supplementary material for: A protocol for a systematic review and meta-analysis of the effect of muscle energy techniques on shoulder joint pain
Source: PLoS One. 2025 Apr 17;20(4):e0321176. doi: 10.1371/journal.pone.0321176 (PMC12005804; doi:10.1371/journal.pone.0321176)
Supplement: Attachment — Submitted filename: Response to Reviewers.docx [file pone.0321176.s005.docx]

**Response to Reviewers**

Dear Editor and Reviewers,

We sincerely appreciate the valuable comments and suggestions provided by the reviewers, which have significantly helped us to improve the quality of our manuscript. We have addressed each of the reviewers’ comments in detail below. Furthermore, we would like to inform you that the entire manuscript has been professionally edited by Editage, a reputable language editing service, to ensure clarity and accuracy in English language usage. We have also received an official certificate from Editage to verify this process, which we are prepared to submit if required.

**Response to Reviewer #1**

**Comment 1:** “Studies selected for systematic review and meta-analysis will include randomized controlled clinical trials, quasi-randomized controlled trials, and controlled (nonrandomized) clinical trials” (lines 61 and 62) needs to be corrected, as well as the text in Table 1 (Research method: Randomized controlled clinical trials, quasi-randomized controlled trials, nonrandomized controlled clinical trials, except for qualitative research and case studies).

**Response:** We have made the requested corrections to the text in both lines 61-62 and Table 1. In the revised manuscript, we have clarified that only randomized controlled trials will be included, as per the latest inclusion criteria. Non-randomized controlled trials have been excluded from our review criteria to ensure methodological rigor.

**Comment 2:** Improve the new text of the inclusion and exclusion criteria in lines 134 and 135.

**Response:** We have refined the wording of the inclusion and exclusion criteria in lines 134 and 135 to enhance clarity and precision. The updated criteria explicitly describe the type of studies included and excluded to align with our study objectives and methodology.

**Comment 3:** There are word errors throughout the manuscript, and the quality of the scientific writing needs to be improved. It is suggested that the entire text be reviewed by a native speaker or a professional language editing service.

**Response:** We acknowledge this suggestion and have had the entire manuscript professionally edited by Editage. This editing process has corrected any language errors and improved the overall quality of scientific writing. As mentioned, we have received a certificate from Editage confirming the completion of this professional editing service.

**Response to Reviewer #2**

**Comment 1:** The "point" in the subtitle "Effect of muscle energy techniques on shoulder point pain: a protocol" may be a typo of "joint."

**Response:** We apologize for this oversight. We have corrected the subtitle to "Effect of muscle energy techniques on shoulder joint pain: a protocol."

**Comment 2:** You said you no longer included non-randomized clinical trials, but you didn't delete "controlled (non-randomized) clinical trials" from the methods section of your ABSTRACT.

**Response:** Thank you for pointing this out. We have revised the methods section of the abstract to exclude any mention of non-randomized clinical trials, aligning it with the revised inclusion criteria.

**Comment 3:** Appropriate simple descriptions of the expected research results can be added in the abstract, such as what level of evidence support is expected to be provided for the application of MET techniques in the treatment of shoulder joint pain through the research.

**Response:** We appreciate this suggestion. We have added a brief description in the abstract regarding the anticipated impact of our study, specifically mentioning the potential contribution of evidence supporting the use of MET techniques in treating shoulder joint pain.

**Comment 4:** Now it is October 2024, and it is not appropriate for "comprehensively search the following databases from their inception up to April 2024". Please modify it.

**Response:** This has been updated to reflect the current time frame, with the revised text stating that the search will be conducted up to October 2024.

We hope that the revisions and explanations provided here satisfactorily address all the comments and suggestions from the reviewers. We appreciate the opportunity to improve our manuscript and thank the reviewers again for their constructive feedback.

Best regards,
Ye Ji Kim
