# Supplementary material for: A protocol for a systematic review and meta-analysis of the effect of muscle energy techniques on shoulder joint pain
Source: PLoS One. 2025 Apr 17;20(4):e0321176. doi: 10.1371/journal.pone.0321176 (PMC12005804; doi:10.1371/journal.pone.0321176)
Supplement: Attachment — Submitted filename: Response to Reviewers V2.docx [file pone.0321176.s006.docx]

**Response to Reviewers**

Dear Editor and Reviewers,

We sincerely appreciate the valuable comments and suggestions provided by the reviewers, which have significantly helped us to improve the quality of our manuscript. We have addressed each of the reviewers’ comments in detail below.

**Response to Reviewer #2**

**Comment 1:** Reviewer #2: The article has been revised very well. Thank you for your efforts. There is still one point that needs to be improved.

Supplement of Long-term Follow-up and Safety Assessment: The current research protocol mainly focuses on the short-term efficacy of MET on shoulder joint pain and function, but lacks consideration of its long-term effects. A plan for long-term follow-up of patients should be added. For example, the indicators such as pain, range of motion of the joint, and quality of life should be evaluated at 3 months, 6 months, or even 1 year after treatment to comprehensively understand the durability of the efficacy of MET. At the same time, the safety assessment during the MET treatment process is not involved in the article. Observation indicators and methods should be supplemented to record possible adverse reactions (such as muscle strains, excessive joint movement, etc.) to ensure the safety of this treatment method in clinical application.

**Response:** We added an evaluation tool, including safety assessment, during the treatment process in lines 169-183. Additionally, in line 198, we added a statement that side effects of the intervention should also be collected during intervention data collection. In lines 201-203, a sentence was added, stating that in order to consider long-term effects, the results of the intervention should be evaluated at 3 months, 6 months, and 1 year.

We hope that the revisions and explanations provided here satisfactorily address all the comments and suggestions from the reviewers. We appreciate the opportunity to improve our manuscript and thank the reviewers again for their constructive feedback.

Best regards,
Ye Ji Kim
